# Mutations in Prion Protein Gene: Pathogenic Mechanisms in C-Terminal vs. N-Terminal Domain, a Review

**DOI:** 10.3390/ijms20143606

**Published:** 2019-07-23

**Authors:** Livia Bernardi, Amalia C. Bruni

**Affiliations:** Regional Neurogenetic Centre, ASP Catanzaro, 88046 Lamezia Terme (CZ), Italy

**Keywords:** Prion protein mutation (PrP mutation), PrP N-terminal domain, PrP C-terminal domain, interdomain *cis* interaction, Proline, *PRNP* gene, dementia

## Abstract

Inherited mutations in the Prion protein (PrP), encoded by the *PRNP* gene, have been associated with autosomal dominant neurodegenerative disorders, such as Creutzfeldt–Jacob disease (CJD), Gerstmann–Sträussler–Scheinker syndrome (GSS), and Fatal Familial Insomnia (FFI). Notably, *PRNP* mutations have also been described in clinical pictures resembling other neurodegenerative diseases, such as frontotemporal dementia. Regarding the pathogenesis, it has been observed that these point mutations are located in the C-terminal region of the *PRNP* gene and, currently, the potential significance of the N-terminal domain has largely been underestimated. The purpose of this report is to review and provide current insights into the pathogenic mechanisms of *PRNP* mutations, emphasizing the differences between the C- and N-terminal regions and focusing, in particular, on the lesser-known flexible N-terminal, for which recent biophysical evidence has revealed a physical interaction with the globular C-terminal domain of the cellular prion protein (PrP^C^).

## 1. Introduction

Prion diseases, also known as transmissible spongiform encephalopathies, are progressive, fatal, neurodegenerative disorders based on the misfolding of the prion protein [1]. They can affect both humans and a wide variety of animals, including sheep, goats, bovine, mule deer, and elk [1]. Creutzfeldt–Jakob disease (CJD), fatal familial insomnia (FFI), and Gerstmann–Sträussler–Scheinker syndrome (GSS) constitute the more common and diverse human phenotypes of prion diseases [1]. These phenotypes exhibit different characteristics based on the onset/duration of the disease, the clinical manifestations, neuropathological changes, transmissibility, and molecular features of scrapie-like prion protein (PrP^Sc^) [2,3]. According to their etiology, human prion diseases can be divided into three groups: (1) sporadic CJD, that constitutes 85–90% of CJD cases; (2) diseases acquired by infection by external prions, such as in the case of Kuru, iatrogenic CJD, and variant CJD (2–5% of CJD cases); and (3) diseases caused by a genetic mutation in the prion (*PRNP*) gene, such as in the case of familial CJD, genetic CJD, GSS, and FFI (approximately, 10–15% of all prion diseases), with an autosomal dominant inheritance pattern [4]. However, the real incidence of the dominantly inherited prion diseases (IPD) is not fully known, since familial clusters have not been systematically recognized or reported [5].

A key event in the pathogenesis of infectious, sporadic, and IPD is the misfolding of the normal form of the prion protein, PrP^C^, into the typically protease-resistant-sheet rich isoform, defined as the scrapie prion protein (PrP^Sc^), by a conformational rearrangement. The PrP^Sc^ constitutes the transmissible agent (“prion”), able to recruit and convert natively folded PrP^C^ into de novo PrP^Sc^ via an autocatalytic process [6,7].

In the case of infectious and sporadic prion diseases, the normal prion protein PrP^C^ undergoes conformational changes into the self-propagating, misfolded PrP^Sc^ conformer. Conversely, in the inherited form of the disease, an alteration in the conformation of PrP^C^ may be induced by a genetic mutation in the *PRNP* gene, but, despite the misfolding of the prion protein (PrP) also playing a central pathogenic role, the process by which *PRNP* mutations promote the development of self-propagating conformations has not been completely elucidated [8]. The precise physiological function of PrP^C^ is largely unknown; however, it appears to be concentrated primarily at pre- and postsynaptic neuronal membranes [9] and the well-documented ability of PrP^C^ to coordinate Cu^2+^ and Zn^2+^ suggests it plays a role in metal ion homeostasis [9,10]. In structural terms, the mature PrP^C^ protein (residues 23–231) is composed of an independent and flexible N-terminal region (residues 23–120) and a C-terminal globular domain (residues 121–231), which physically interact with each other [11]. To date, little is understood about the disordered N-terminal domain, the importance of which has largely been overlooked, because known pathogenic mutations in this region have been shown to have no effect on the structure, stability, or dynamics of native mouse prion protein [12]. However, this domain is involved in the determination of the physical properties of disease-related forms of PrP—the high degree of conservation between species of this flexible domain probably reflects a strong functional significance, and this flexibility has diverse biological endpoints [11]. Indeed, pathogenic mutations, such as the G113V and A116V, in the N-terminal domain, may induce prion pathogenesis by accelerating misfolding and aggregation, modifying the structure in the palindromic region, which appears to be a site for intermolecular association in the oligomers [12]. To date, most of the known *PRNP* pathogenic mutations have been identified in the C-terminal domain. Recently, a missense P39L mutation in N-terminal domain of the prion protein was reported by several authors, in patients affected by frontotemporal lobar degeneration (FTLD) syndrome, which were negative for mutations in genes causative of dementia [13,14,15]. Given all these data, the purpose of our report is to provide updated insights into the pathogenic mechanisms of *PRNP* mutations, emphasizing the differences between the C- and N-terminal domains and focusing in particular on the lesser-known flexible N-terminal, for which recent biophysical evidence has revealed a physical interaction with globular C-terminal domains of PrP^C^ [10].

## 2. The Function of the Prion Protein

To date, the exact physiological role of PrP has not yet been definitively clarified. The expression of the wild type PrP is diffuse in neurons, neuroendocrine cells, and stromal cells of the lymphoreticular system. The highest levels have been observed in the central nervous system in the synaptic membrane. The determinant step in prion infection is the conversion of the conformation of PrP^C^ into a protease-resistant β-sheet, PrP^Sc^ [2], with concomitant expression of PrP^C^, which is required and rate-limiting [16].

The PrP is bound to the outer membrane of the cell surface, in specific “rafts” (cholesterol- and glycosphingolipid-rich lipid sites) [17], by a glycosylphosphatidylinositol (GPI) anchor [18,19]. The N-terminal signal peptide (the first 22 amino acids of the precursor protein) is cleaved after translocation across the endoplasmic reticulum membrane [17]. The function of the physiological PrP seems to be to protect against programmed cell death [20]. The PrPC N-terminal domain binds both copper and zinc in vivo and participates in metal ion homeostasis [21]. Cu^2+^ and Zn^2+^ ions coordinate to the N-terminal PrP differently—Cu^2+^ interacts with the octarepeat domain, residues 60–91 with the sequence (PHGGGWGQ) [22,23], and also with residues His96 and His111 [24], whilst Zn^2+^ binds to the octarepeat domain, in which all four histidine residues coordinate a single Zn^2+^ ion [24].

PrP^C^ is a copper-binding protein showing superoxide dismutase activity, appearing to protect against oxidative damage [25] and acting as a cell-surface receptor for signal transduction [26]. Several studies have demonstrated that the mammalian PrP^C^ protein is extremely versatile, involved in proliferation, differentiation, cell adhesion, and synaptic plasticity [27]. Several functions of the PrP^C^ protein depend on its interaction with extra- and intra-cellular signaling partners (ligands). Among these ligands, those found to be advantageous to the cell [27] are laminin and glycosaminoglycans (GAGs), involved in neuronal differentiation and axon growth [28], and neuronal adhesion proteins, such as N-CAM12 that contribute to neurite outgrowth [28].

## 3. The *PRNP* Gene, Mutations, and Inherited Prion Diseases

*PRNP* (NC_000020.11), located on chromosome 20 (4686151-4701588), is a 16 Kb long gene, containing two exons. Exon 1 has the role of a transcriptional initiation site, whereas the open reading frame (ORF) encoding the PrP protein, composed of 253 amino acids, is located in the exon 2 [29]. Different mutations have been reported as causative for diseases, but their effects have been associated with a variety of heterogeneous phenotypes [29]. Pathogenic mutations in the ORF of the *PRNP* are the only known causes of IPD [2]. These fatal neurodegenerative disorders follow a dominant mode of inheritance and are traditionally classified clinically as CJD, GSS, and FFI [30]. *PRNP* mutations consist of point mutations leading to an amino acid substitution or a premature stop codon, and insertions/deletions of additional (more than three additional) octapeptide repeats (OPRI/OPRD) in the region between codons 51–91 of the PrP that encodes a 5-mer repeat region consisting of a nonapeptide followed by four identical octapeptides. The frequency and distribution of these mutations differ between Europeans and East Asians [31]. Some pathogenic *PRNP* mutations are typically associated with particular clinical categories of prion disease [31], conferring the diagnosis of IPD and sub-classification according to a specific mutation (Table 1). Other mutations are involved in a spectrum of clinical and pathological phenotypes that vary across and within families carrying the same genetic alteration [32], often with striking phenotypic heterogeneity. In addition, different *PRNP* gene mutations have been suggested to play a potential role in clinical pictures mimicking other neurodegenerative diseases, such as Frontotemporal dementia (FTD) [33,34,35,36,37,38], Cerebral amyloid angiopathy (CAA) [5], familial neuropsychiatric illness [39], familial Alzheimer’s disease (AD) [40], and Huntington’s disease [41]; whereas the clinical picture may not be specific or confined to psychiatric features [32]. Of note, the most prevalent missense mutations causing IPD and a series of Single Nucleotide Polymorphisms (SNPs) are localized in the C-terminal domain. Conversely, in the N-terminal region between codons 51–91 (the region consisting of the octapeptide repeats), only OPRI/OPRD are found as polymorphisms and pathogenic mutations. The presence of any pathogenic point mutation in residues 23–50 remained unknown until the description of the missense Pro39Leu mutation, reported in two patients affected by FTLD syndrome [13] and successively in another FTD patient [14], in which all three patients were negative for mutations in other known causative genes. Pro39Leu is the first mutation described in the N-terminal domain located in a codon (the 39 codons), before the known 102 residue (pathogenic mutation Pro102Leu causative of GSS) [15]. Nevertheless, functional studies to determine whether and how the Pro39Leu mutation may exert its pathogenic effects still remain to be implemented. Recently, a *PRNP* mutation was described in a young GSS patient, presenting a particular clinical picture with status epilepticus at the age of 34, prefaced by night terrors at age 26, memory problems, behavioral changes and parasomnias subsided after a six-year period, emerged at this age [42]. This mutation consists of a LGGLGGYV insertion (a partial internal duplication) located at the junction between the hydrophobic region of the N-terminus and the globular domain. A subsequent study [43], involving animal modeling, defined a novel misfolded form of mutant PrP^C^ that prefigures the PrP mutated fragment pathognomonic for end-stage GSS with multicentric amyloid plaques [44] that might also be shared by other forms of GSS, thus providing a potential explanation for the early disease onset of the proband.

In addition to these mutations, that appear fully penetrant, many common single nucleotide polymorphisms (SNPs) have also been detected in the ORF of the *PRNP* gene [45]. The most important are the SNPs at codon 129, which have a critical role in susceptibility and as a modifier of prion disease, and alterations in the number of repeats, with up to three additional repeats. This specific genotype of the *PRNP* Met129Val SNP is responsible for the diagnosis of FFI or GSS (Table 1) when associated with the *PRNP* Asp178Asn mutation. Specifically, the Asp178Asn mutation accounts for FFI together with the 129Met genotype, whereas the same mutation associated with the 129Val genotype has been found in CJD (Table 1). Furthermore, the Met129Val SNP seems to be accountable for the phenotypic heterogeneity, such as variance in the age of onset (20–85 years) [32]. Other naturally occurring *PRNP* polymorphisms, such as the Gly127Val [46] and the Glu219Lys [47], completely prevent prion disease. In fact, it has been reported that the Gly127Val SNP in the heterozygous state was subjected to positive evolutionary selection during the epidemic of Kuru (an acquired prion disease epidemic of the Fore population in Papua, New Guinea), providing strong protection against the disease [46]. The Glu219Lys is also a *PRNP* SNP well-known for its protective effects against sporadic CJD [47], and the equivalent substitution in mouse PrP (Gln218Lys) is also protective against mouse-adapted scrapie [48]. It is possible that these effects depend on the inability of Glu219Lys to transform into PrP^Sc^ and on its dominant-negative inhibition of the coexisting wild-type PrP [49].

## 4. The Structure of the N-Terminal and C-Terminal Domains

As for the structure of the PrP, the mature protein (residues 23–231) is composed of two independent structures, the N-terminal (23–120) and the C-terminal domains (residues 121–231). The N-terminal region is a flexible, random coil presenting with a disordered amino acid sequence, whereas the C-terminal region forms a rigid globular domain [45]; it contains a bundle of three α-helices, a short, two-stranded, antiparallel β-sheet, and is stabilized by a disulfide bridge and includes two variably occupied N-linked glycosylation sites. These elements are located in two halves, β1–α1–β2 and α2–α3, which are assembled in the hydrophobic core [50]. The structure of this protein has been conserved during evolution across vertebrate classes, showing a high degree of amino acid sequence similarity [1]. Insertions and deletions are the most common variants detected in the N-terminal of the PrP (amino acid residues 23–90); whereas, in the C-terminal portion (91–231) point mutations are more common. A high degree of sequence conservation has been identified in the N-terminal region between amino acid residues 23–90 and the regions located upstream of the alpha helices 1 and 3 [1]. This domain contains the metal-binding octarepeat (Cu2^+^- and Zn^2+^-binding octarepeat domain, OR) domain, as well as two polybasic charged clusters, and a hydrophobic linker domain.

### 4.1. The C-Terminal Domain: Mechanisms Causing a Conformational Change of PrP in Mammalians

Despite important advances in the last decade, how *PRNP* pathogenic mutations are involved in generating a misfolded PrP remains not clarified and how pathogenic mutations in *PRNP* cause prion disease has yet to be solved. However, study efforts about the mechanism involved in this conformational rearrangement of this protein have indicated that the variation of the *PRNP* sequence by pathological mutations is sufficient to generate prions [45]. Genetic variations in the *PRNP* gene were found mostly in the β2–α2-loop region and in the α2–α3 inter-helical interfaces, which are assembled against each other in the hydrophobic core. Experimental data suggested that the conformation of the β2–α2-loop plays a role in the transmission of prion disease and its susceptibility. Mammals carrying a flexible β2–α2 loop are easily infected by prions, while in animals carrying a rigid loop, prions are poorly infected [51]. Notably, the horse, rabbit, dog, and buffalo are mammalian species reported as resistant to infection from prion diseases isolated from other species [52,53,54,55]. 

PrP structures are characterized by a rigid β2–α2 loop and by a closer contact between the loop and the α3 helix [51]. Thus, it appears that prion resistance is determined by the amino acid sequence of the β2–α2-loop and its long-range interactions with the α3 helix in the C-terminal end. Using molecular dynamic (MD) simulations of some *PRNP* mutations, the mutant structures in aqueous solution have been investigated [56]. Structures of Gln212Pro and Val210Ile mutants show the interruption of aromatic and hydrophobic interactions between the residues located at the interface of the β2–α2 loop and the C-terminal end of the α3 helix. The increased distance between the β2–α2-loop and the α3 helix in the mutants results in higher exposure of hydrophobic residues to the solvent. Glu200Lys, Phe198Ser, and Asp178Asn mutations present similar characteristics. These results indicate that the disorder in the structure of the β2–α2-loop with the loss of contact between the loop and the α3 helix are critical epitopes responsible for the conversion to PrP^Sc^. Indeed, the regions involved in the pathogenic conversion of PrP^C^ to the scrapie form of the protein appear to be the same as affected by disease-linked mutations in terms of structure and flexibility [31]. In fact, the variation in flexibility of the PrP protein mainly involves residues 165–175 and residues 185–200, involving the β2–α2-loop and the α2–α3 structural regions, respectively [57]. The flexibility in the variation facilitates the access to alternate conformational states of the protein, remodeling the sites for molecular recognition events (i.e., protein-protein and protein-ligand interactions) [57]. Molecular dynamics studies have revealed that in rabbits, dogs, horses, and buffalo [52,53,54,55], species resistant to infection from prion diseases, there is a strong salt bridge Asp178-Arg164 (O-N) keeping the β2–α2 loop closely linked and contributing to the structural stability of prion protein. Another recent study about the low prion susceptibility of canids, based on the amino acid sequence of the canine PrP, identified the relevance of the Asp163 amino acid in proneness to protein misfolding, showing it was a key amino acid with characteristics responsible for the high resistance to prion disease [58]. Using in vitro and in vivo models, Fernadez–Borges et al. demonstrated that the presence of this Asp163 residue confers resistance to prion infection when introduced to susceptible animals. Despite the large number of studies to date, the significance of the β2–α2-loop on transmission efficiencies has not been completely clarified [59]. 

### 4.2. The N-Terminal Domain

The importance of the N-terminal region has largely been underestimated because it does not appear involved in prion replication. Nevertheless, it has been shown that this domain is involved in fibrillation and the determination of the physical properties of disease-related forms of PrP [57]. The N-terminal region is a flexible and largely disordered structure. Furthermore, the high degree of conservation between species of segments of this flexible domain, such as residues 23–90, is significant, probably reflecting functional importance [57]. 

Many functional advantages to intrinsically disordered proteins/regions (IDPs/IPRs) are due to the lack of stable tertiary and secondary structures. These advantages are represented by the disorder-to-order transition, increased binding rate malleability of interaction with different partners (binding promiscuity), and specific low-affinity binding. These characteristics of disordered, unstructured proteins, which constitute the basis of modulation of post-translational modifications, such as phosphorylation, acetylation, acylation, carboxylation, glycosylation, methylation, hydroxylation, etc. Post-translational modifications, involving low affinity and high-specificity interactions between a protein and a specific ligand and associated with IDPs and IPRs are especially important for signaling and regulation of the cell (i.e., transcription, DNA repair, signal transduction, autophagy, etc.) [60]. The capability of the PrP^C^ protein in the interaction with multiple extra- and intra-cellular signaling partners (ligands) is due to the structural disorder of the N-terminal domain, which depends on its specific conserved, and not random, amino acid sequence [27]. The susceptibility to prion diseases could be caused by numeric variability and conformational changes observed in this sequence. In the PrP, the N-terminal residue is associated with PrP^C^ internalization [61], for which the initial polybasic region (amino acids 23–28 NH2-KKRPKP) has been shown to be significant [61]. Moreover, the N-terminal domain (amino acids 23–90) acts as a raft-targeting signal, as it is sufficient to confer raft localization when fused to a non-raft transmembrane-anchored protein [61]. The polybasic region including amino acids 23–30, seems crucial for the correct folding of the PrP^C^, and may also regulate the acquisition of the strain-specific conformations in the disease [61]. The region including amino acids 23–50 confers a cellular protective effect resulting in reduced intracellular reactive oxygen species (ROS) levels [62].

### 4.3. Natural Ligands of the N-Terminal Domain

Natural ligands promote structural rearrangements and play a significant role in the modulation and stabilization of the structure of proteins. These proteins undergo, during the course of their biological function, several types of conformational changes, which are responsible for interactions between proteins and low molecular-weight ligands or larger macromolecules. The structural transformations induced by a ligand in a protein can vary, ranging from a negligible decrease in the conformational stability to complete protein unfolding [60,63]. The flexible unstructured N-terminal region provides the PrP^C^ with several advantages. The extended linear protein region may allow interaction with many ligands ranging from small molecules (e.g., Cu^2+^, Zn^2+^) [10,64] to macromolecules (e.g., phospholipids, proteins); however, the disordered proteins and their advantages have yet to be described. Natural binding ligands along the entire extent of the PrP^C^ molecule are represented by lipids, nucleic acids, and glycosaminoglycans, which confer to the protein diverse, and sometimes contrasting, activities [65,66,67,68,69,70].

Different studies have shown that the N-terminus of PrP can interact with a broad range of ligands: (1) Metal ions (such as Cu^2+^ and Zn^2+^), which bind to the amino acid residues 59–90, demonstrate the involvement of this region in copper endocytosis and metabolism [10]. Indeed, some studies have shown that prion proteins with insertion variants in the N-terminal region have altered conformation, increased ligand binding activity, and are more susceptible to oxidative attack [10]; (2) Aβ oligomers with high affinity, mediate neurotoxic effects, being the polybasic stretch at the extreme N-terminus of the two critical regions for the interaction [10]; (3) Tubulin, interacts with PrP regions mapped to the N-terminus of PrP spanning residues 23–50 and 51–91. The PrP octapeptide repeats are critical for this binding activity, given that binding becomes stronger as the number of octapeptide repeats increases, thus suggesting a potential role for PrP in the regulation of the microtubule dynamics in neurons [71]; (4) Acetylcholinesterase (AChE), a key protein in the cholinergic system both in neural and non-neural tissues, through a heterologous association, induces aggregation of monomeric PrP and modifies the structural properties of PrP amyloid fibrils. The PrP-AChE interaction occurs at two sites in the PrP N-terminal domain (residues 23–99 and 100–120) [72]; (5) Melanin, a main determinant of skin color that interacts with PrP at the N-terminal domain specifically, strongly interacts with the PrP region at amino acids 23–50 and weakly interacts with the PrP octarepeat peptide region including residues 51–90; the pathogenic role of the PrP-melanin interaction remains undefined, even if this skin pigment might be useful for evaluating the functions of other ligands at the N-terminal region [73]; and (6) Nucleic Acids, including RNA and DNA, have been shown to interact with PrP both in vitro and in vivo, indicating their involvement as molecular cofactors of PrP^C^ conversion into PrP^Sc^-like species [74].

## 5. Pathogenic Mechanisms

### 5.1. The N-Terminal Domain Is a Toxic Effector Regulated by the C-Terminus

To date, the N-terminal and C-terminal domains have often been considered as independent and non-interacting units. Cellular and biophysical studies have demonstrated that this scheme cannot be correct, and rather, the PrP^C^ consists of two functionally distinct modules, with the globular domain and the flexible tail-exerting regulatory and executive functions, respectively [75]. In fact, it has been reported that the flexible N-terminal tail is required to transmit toxic signals that originate from the globular domain and trigger oxidative stress and calpain activation [75]. An interesting study evaluating the PrP^C^ N-Terminal domain in prion species barriers examined the role of amino acids 23–90 in cross-species conversion using real-time, quaking-induced conversion (RT-QuIC) to model the central molecular event in prion disease, i.e., the template misfolding of the PrP^C^ to the pathogenic isoform. By comparing the conversion efficiency of various prion seeds in either full-length (amino acids 23–231) or truncated (amino acids 90–231) PrP^C^, it was observed that, in addition to the primary sequence, prion species barriers are controlled by interactions of the N-Terminal domain with PrP^C^ [76].

In another study, it was observed that deletion of the *PRNP* gene was tolerated both in cells and in transgenic animals. Similarly, the deletion of the N-terminal domain (residues 23–124) resulted to be benign [10]. Instead, some internal deletions within the PrP^C^ N-terminal domain have been reported to induce varying degrees of neurotoxicity in transgenic mice, and the severity of the neurotoxic phenotype depended on the length of the amino acid deletion. Deletions in residues spanning 105–125 at the end of the N-terminus domain produce spontaneous neurodegeneration similar to that of natural prion diseases but without accumulation of PrP^Sc^ [77]. These phenotypes are suppressed in a dose-dependent manner by the co-expression of the normal PrP, suggesting that the normal and deleted molecules interact with each other, or compete for binding to a common molecular target, affecting both physiological and pathological functions. The D105–125 shortest deletions (DCR, for the central region), produces the most severe neurodegenerative phenotype and requires the largest amount of normal PrP for rescue [77]. Recently, Wu et al. [78] discovered that these deleted forms of PrP induce large, spontaneous ionic currents when expressed in a variety of cell lines and primary neurons. These ionic currents could be silenced by co-expression of the wild-type PrP in the same cells, similar to the rescuing effects of wild-type PrP in transgenic mice expressing deleted PrP. Thus, it appears that the spontaneous ionic currents themselves, or a closely associated phenomenon, may play a role in the neurodegenerative phenotype observed in these mice. The authors showed that the expression of only the N-terminal domain, without the C-terminal domain, induced spontaneous currents, which suggested that for PrP^C^, the N-terminal domain may act as a neurotoxic effector whose activity is regulated by its C-terminal domain. Thus, this interaction involving the N- and C-domain may regulate the physiological activity of PrP^C^, and the disruption of this interaction could play a role in the pathophysiology of neurodegenerative disorders [78].

### 5.2. Important Key Elements in the Pathogenic Mechanisms: Metal Ions Cu^2+^ and Zn^2+^ and the Proline Amino Acid

#### 5.2.1. Cu^2+^ and Zn^2+^ Promote Interdomain Interaction in *cis*

Among the known ligands of the N-terminal domain, there has been substantial effort to better understand the role of metal ions in normal PrP^C^ physiology and in prion disease pathogenesis. To date, growing evidence supports the concept that the physiological function of PrP^C^ is associated with its metal-binding properties [79]. The octarepeat-bound Cu^2+^ and Zn^2+^ promote an interdomain interaction in *cis*, in terms of an association of the PrP^C^ N- and C-terminal domains [10]. This domain–domain *ci*s interaction leads to sequestration of the N-terminal domain and regulation of cell surface receptor interactions via an autoinhibitory-like mechanism, with the direct involvement of metal ions [10]. However, this autoinhibitory effect is not due to direct binding of Cu^2+^ or Zn^2+^ ions but is attributed to the long-range tertiary interactions involving the N-terminal domain, given that the interdomain *cis* interaction is stabilized by the coordination of Cu^2+^ and Zn^2+^ ions [10]. In a recent paper, Eigenbrod et al. [64] established new transgenic mouse lines expressing PrP with disrupted copper-binding sites within all four histidine-containing ORs (sites 1–4, H60G, H68G, H76G, H84G, “TetraH>G” allele) or at site 5 (composed of residues His-95 and His-110; "H95G" allele) and monitored the formation of misfolded PrP in vivo [64]. The authors concluded that the above OR substitutions influenced the *cis* interactions between the OR region, while disruptions of the site 5 region influenced pathogenic outcomes by impacting on the PrP globular C-terminus domain [64].

#### 5.2.2. Role of the Proline Amino Acid in the PrP Protein

Proline is a nonpolar, non-essential, cyclic amino acid, and imparts a degree of the structure onto proteins due to the steric constraints of the rigid pyrrolidine ring [80]. Proline represents a disruptor within regular secondary structure elements such as α-helices and β-sheets. Multiple prolines and hydroxyprolines in a row can create a polyproline helix, the poly(L-proline) II (PPII) helix, which is the predominant secondary structure in proteins with high conformational flexibility, such as collagen. The presence of a proline in the peptide gives it its special features like elasticity and tensile strength. The hydroxylation of proline, by prolyl hydroxylase in a hydroxylation reaction, increases the conformational stability of collagen significantly, being the hydroxylation of proline critical for maintaining the connective tissue of higher organisms. Proline plays a key role in molecular recognition, particularly in intracellular signaling. The domains rich in proline form “pockets” interacting with ligands, which are critical for intracellular signal transduction. Proteins with high proline concentration are directly involved in signal transduction. Studies using amino acid substitutions to perturb OR rigidity, have allowed to deduce that the mechanism involved in the pathogenesis may be as a consequence of altered OR rigidity, caused by pleiotropic effects of proline substitutions that limit OR flexibility, showing that the N-terminal domain serves as a cell surface scaffold to bind diverse macromolecules and co-factors [11,81].

### 5.3. PRNP Mutations and Disruption of cis Interaction as a Mechanism of Neurotoxicity

**Figure 1:**
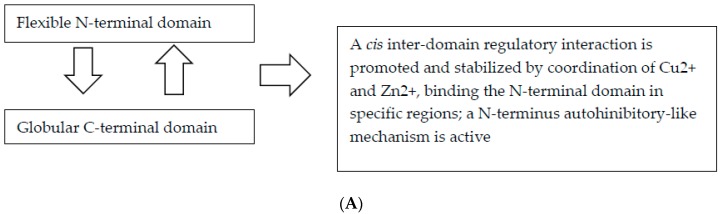

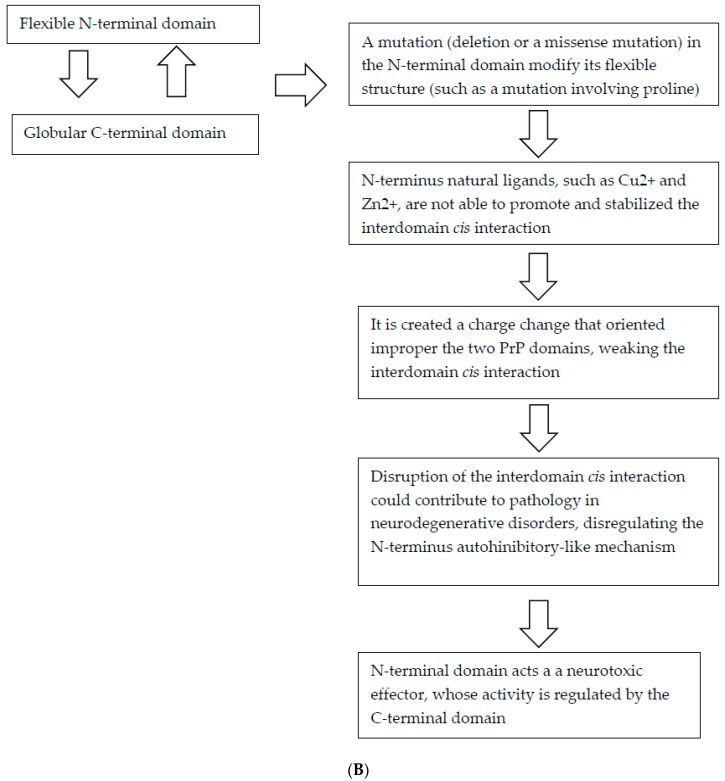
Flow chart depicting feedback loops involved in the physiological and pathological function of PrP. (**A**) The physiological function of PrP; (**B**) The hypothesized pathological mechanisms influencing the function of PrP.

The majority of the PRNP pathogenic mutations causing CJD, FFI, and GSS, result in amino acid substitutions in either the C-terminal domain of the PrP^C^, or in the linker region that separates the C-terminal from the N-terminal domain [32]. Interestingly, many of the pathogenic mutations of the C-terminal domain have the effect of decreasing the negative charge of the domain [10], resulting either in a gain of a positive charge, loss of a negative charge, or both [10]. Biophysical evidence supporting the role of the electronegative C-terminal pocket in the *cis* interaction with the N-terminal domain, suggests that the human mutations responsible for familial CJD (i.e., E200K and D178N) and either CJD or FFI based on the codon at residue 129), respectively, showed weakened *cis* interactions. Otherwise, the GSS-associated Pro102Leu mutation in humans resulted in a mild weakening of the strength of the *cis* interaction, indicating that the conformational rigidity of the proline at this codon may be involved in correctly orienting the two PrP domains for suitable *cis* interactions. A similar pathogenic mechanism could be shared from all mutations involving proline, including the recently published Pro39Leu (Figure 1).

### 5.4. Pathogenic Mutations within the N-terminal Disordered Palindromic Region of PrP Accelerate the Formation of Misfolded Oligomers

A recent study [12] reported that two pathogenic mutations in the palindromic region of the N-terminus (G113V and A116V), have no effect on the structure, stability, or dynamics of native mouse PrP, but accelerate the formation of misfolded oligomers with a high degree of neurotoxicity. This palindromic sequence, spanning residues 111–120 (VAGAAAAGAV), appears to be a site for inter-molecular association in the oligomers, and plays a role in the assembly of fibrils, and in the structural changes accompanying prion conversion [82]. Moreover, this segment seems to be essential for the productive association of PrP^C^ with PrP^Sc^, which leads to prion propagation in animals [83]. 

## 6. Conclusions

In this paper, we reviewed the PrP protein structure, *PRNP* gene mutations, and the latest evidence from recent structural and biophysical studies. These studies revealed the importance of physical interdomain *cis* interactions between the N- and C-terminal regions of PrP^C^ stabilized by Cu2+- and Zn2+ binding to the N-terminal OR. This interaction in the PrP^C^ may represent a molecular mechanism involved in the regulation of the activity of the N-terminal domain and provides insights into the biochemical mechanisms induced by many pathogenic *PRNP* mutations found in both the C- and N-terminal domains. In particular, mutations located in the N-terminal domain involving proline, such as the Pro39Leu, for which functional and neuropathological studies are unfortunately not currently available, could act through this pathogenic mechanism. Taken together, we might consider the N- and C-terminal domains equally important from the standpoint of pathogenic mechanisms of causative mutations of *PRNP* found in inherited prion diseases. Novel mutations [84,85] (Table 1), with unclear pathogenicity (such as G127S, N171S, P238S, [31]) or associated with clinical phenotypes different from typical prion diseases [86], should be carefully studied, in particular, in view of these recently described pathogenic mechanisms. Further specific pathological, biochemical, and molecular studies are warranted [87] to investigate how variations in these domains might trigger the extreme phenotypic variability associated with the PrP protein, in terms of the pathogenicity towards neurodegeneration and not towards the specific typical prion diseases.

## Figures and Tables

**Table 1:** *PRNP* mutations and associated phenotypes [13,14,29,31].

Mutation	Domain	Clinical Phenotype
Pro39Leu	N-terminal	FTLD, FTD
Pro102Leu	N-terminal	Classical CJD-like symptoms, GSS
Pro105Leu	N-terminal	GSS, spastic paraparesis and progressive dementia
Pro105Ser, Pro105Thr	N-terminal	GSS
Gly114Val	N-terminal	CJD, neuropsychiatric symptoms
Ala117Val	N-terminal	CJD, Progressive cortical dementia and cerebellar ataxia
Octapeptide insertions (from 4 to 9 OR insertions)	N-terminal	CJD
Gly131Val	C-terminal	GSS, tremor and apraxia
Gln160-nonsense; Tyr163-nonsense	C-terminal	Alzheimer’s disease-type pathology
Val176Gly	C-terminal	Cerebellum ataxia, personality changes and progressive dementia
Asp178Asn	C-terminal	CJD and FFI depends on the allele on codon 129, Met or Val
Val189Ile	C-terminal	Classical and atypical CJD (behavioral abnormalities, ataxia and extrapyramidal features)
Val180Ile, Thr183Ala, Thr188Lys, Glu196Lys, Glu196Ala, Glu200Lys, Glu200Gly, Val203Ile, Arg208His, Val210Ile, Glu211Gln, Ile215Val	C-terminal	Classical and atypical CJD
Gln160-nonsense, His187Arg, Phe198Ser, Asp202Asn, Glu2011Gln, Gln212Pro, Gln217Arg, Tyr226-nonsense, Gln227-nonsense	C-terminal	Classical and atypical GSS
